# The representation of the verb's argument structure as disclosed by fMRI

**DOI:** 10.1186/1471-2202-10-3

**Published:** 2009-01-15

**Authors:** Ramin Assadollahi, Marcus Meinzer, Tobias Flaisch, Jonas Obleser, Brigitte Rockstroh

**Affiliations:** 1 ExB Communication Systems GmbH, 80333 Munich, Germany; 2 Department of Psychology, University of Konstanz, 78457 Konstanz, Germany; 3 University of Florida, College of Public Health Professions, Gainesville, Florida-32611, USA; 4 Max Planck Institute for Human Cognitive and Brain Sciences, 04103 Leipzig, Germany

## Abstract

**Background:**

In the composition of an event the verb's argument structure defines the number of participants and their relationships. Previous studies indicated distinct brain responses depending on how many obligatory arguments a verb takes. The present functional magnetic resonance imaging (fMRI) study served to verify the neural structures involved in the processing of German verbs with one (e.g. "snore") or three (e.g. "gives") argument structure. Within a silent reading design, verbs were presented either in isolation or with a minimal syntactic context ("snore" vs. "Peter snores").

**Results:**

Reading of isolated one-argument verbs ("snore") produced stronger BOLD responses than three-argument verbs ("gives") in the inferior temporal fusiform gyrus (BA 37) of the left hemisphere, validating previous magnetoencephalographic findings. When presented in context one-argument verbs ("Peter snores") induced more pronounced activity in the inferior frontal gyrus (IFG) of the left hemisphere than three-argument verbs ("Peter gives").

**Conclusion:**

In line with previous studies our results corroborate the left temporal lobe as site of representation and the IFG as site of processing of verbs' argument structure.

## Background

Most verbs describe events with one or more participants [1]. The verb's *argument structure *defines the number and relationships of participants needed for a complete event. For instance, a sentence like "Peter gives Jim a book" includes three participants with three *thematic roles*: the agent (Peter), the recipient (Jim) and the theme (the book; [2]). For a verb like "give" the entry in the mental lexicon must comprise such information in addition to phonetic and orthographic information. Neurolinguists have always been interested whether and how this feature of verbs is represented in the brain, the majority of studies employing entire sentences, wh-questions (e.g. "Where did he go?", "What did he give her?", "Why did he give her the book?"), or sentences including syntactic or semantic violations (e.g. [2-6]. Functional imaging studies disclosed the middle temporal gyrus (MTG) and the inferior frontal gyrus (IFG, BA 45/47) of the left hemisphere [7-9] to be involved in the processing of the verb's argument structure. Additional activity in the left IFG (BA 44/45) was found, when grammatically complex sentences requiring working memory resources [10,11] or when argument hierarchies [3] were processed. Whenever words were presented in the grammatically correct order in one, and out of order in another condition, activation of the left IFG and MTG was *more *pronounced to words in *correct *sentences [12].

In a recent fMRI study, Thompson et al. [13] compared nouns and verbs with increasingly more complex argument structure [one-argument (e.g. sleep) vs. two-arguments (e.g., chase) vs. three arguments (put)] during a lexical decision task. While the processing of all lexical items activated a large bilateral network including the occipito-temporal, superior-inferior parietal and superior temporal areas, only verbs activated left inferior frontal and middle temporal areas. The comparison of verbs revealed more pronounced activity mainly in the left inferior parietal cortex (IPC; angular and supramarginal gyrus) for one-argument relative to two-argument verbs. Additional right hemispheric IPC involvement was found, when one-argument verbs were compared to two- and three argument verbs. Results were interpreted as reflecting the integration of semantic and syntactic information that are more pronounced for verbs that possess more complex argument structure.

In a previous magnetoencephalographic (MEG) study [14] the argument structure of verbs was systematically varied between one and three, while subjects processed the verbs in a lexical decision task. Verbs were presented either in isolation or together with a name, thus, in a minimal syntactic context. Around 250 to 300 ms after stimulus onset isolated one-argument verbs induced the most pronounced activation in the left middle temporal gyrus, followed by two-argument verbs, while three argument verbs provoke the weakest activation. Whenever the same verbs preceded by a proper name, specifying the subject, additional activation between 350 and 450 ms in the left inferior frontal gyrus was larger and peaked earlier for one-argument verbs, which require no further arguments to form a complete sentence. This suggests that the activated areas vary depending on the linguistic context.

On this background the present study aimed at specifying the cortical structures involved in the processing of verbs' argument structures exploiting the spatial resolution power of fMRI. Within a silent reading design the argument structure of verbs was varied between one and three, and maximum activation was expected to one-argument verbs in temporal areas of the left hemisphere. Again, verbs were presented either in isolation or in the minimal syntactic context of a name that filled the first position of the argument structure. Compared to designs employing entire sentences, this comparison should disclose, whether the crucial information about the relationship of participants and events was already retrieved with the verb and its argument structure or whether a minimal context was required. Thereby, the present study should add to the specification of parsing and processing in the absence of full sentences. If the context was required to activate syntactic processing [3,6], this condition should activate areas known to be involved in syntactic processing, that is, the left inferior frontal gyrus [11]. However, the previous MEG study [14] had suggested that already the minimal context of a proper name filling the first argument was sufficient to activate the IFG. Thus, we hypothesized that even the minimal context of a name should automatically start the integration of the name and the verb to a sentence representation. Therefore, we expected brain responses in the left IFG to distinguish the verb-categories in this condition, too.

## Results

Results were obtained from 20 subjects.

### Basic contrasts (verbs versus fixation baseline)

**Figure 1:**
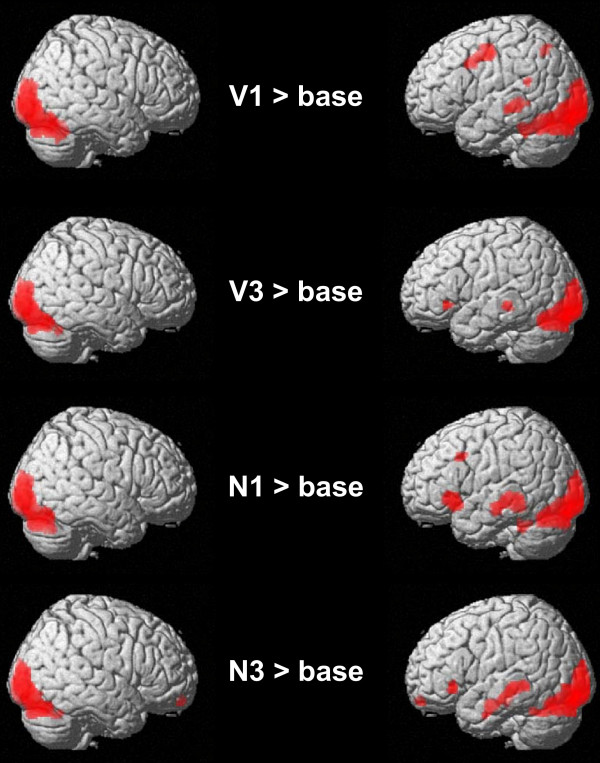
**Shows the general activity pattern elicited by the four experimental conditions compared to the fixation baseline (N1/3 refers to one-/three-place verbs preceded by a name; V1/3 refers to one-/three-place verbs preceded by a senseless letter string)**. Only clusters surviving a threshold of p < .05 family wise error-corrected (FWE) and a cluster extent of k>20 are reported; the voxel threshold was set to p < .001 (uncorrected). Right column = left side of the brain.

The comparison of the four experimental conditions [one-argument or three-argument verbs preceded by a name (N1; N3); one-argument or three argument verbs preceded by senseless letter strings (V1; V3)] with the fixation baseline revealed similar activity in bilateral primary and secondary occipital cortices and in the fusiform gyrus (see Figure 1). The comparison of one-argument verbs presented in isolation and in context (V1 and N1) and of three-argument verbs preceded by a name (N3) disclosed additional activity in the middle temporal gyrus (MTG). MTG activity in response to three-argument verbs presented in isolation was only significant for an uncorrected cluster threshold (p. < 05). Significant activity in the inferior frontal gyrus (BA 47) was restricted to one-argument verbs preceded by a name, thus, creating a complete phrase [Additional file 1 summarizes all contrasts].

### Specific contrasts

When comparing isolated one-argument with isolated three- argument verbs (V1 > V3), more pronounced activity was found in the inferior frontal gyrus of the right hemisphere (BAs 44/9: peak activity x/y/z: 56/8/16; k = 44; Z = 5.4). In the left hemisphere, isolated one-argument verbs induced more activity than three-argument verbs the inferior temporal fusiform gyrus (BA 37, -46/-54/-14, k = 154, Z = 4.1). No significant areas were found for the inverse contrast (V3 > V1). When comparing one-argument verbs in context with three-argument verbs in context (N1 > N3), activity was found only in one left hemispheric cluster (k = 81) that comprised anterior superior temporal (BA 38, -48/15/-9, Z = 4.2) and inferior frontal areas (BA 47, -53/19/-3, Z = 3.8). Again, no significant difference was found for the inverse contrast.

The comparison of verbs with and without context (N1 > V1; N3 > V3) did not disclose differences for one-argument verbs. However, three-argument verbs provoked activation in middle and inferior temporal areas (BA 21, -55/-3/-17, Z = 4.2) of the left hemisphere. Irrespective of the number of arguments, verbs presented in context activated a large cluster that comprised superior/middle and inferior temporal areas (BAs 21/38/20, -53/-5/-15, k = 231, Z = 4.7) and a cluster in the right middle temporal gyrus (BA 21, 55/-1/-18, k = 86, Z = 3.8) relative to verbs presented in isolation (N1/3 > V1/3).

## Discussion

Our previous MEG study [14] had suggested distinct patterns of cortical activity to verbs depending on their argument structure, therefore, the present study served to clarify the localization of involved cortical structures exploiting the enhanced spatial resolution power of fMRI. Confirming the previous results, verb-elicited activity varied with the number of obligatory arguments in the middle temporal gyrus and the inferior frontal gyrus: one-argument verbs "snores" led to stronger activity than three-argument verbs ("gives") in the inferior temporal fusiform gyrus (BA 37) of the left hemisphere. When presented in context of a proper name, one-argument verbs ("Peter snores.") induced more pronounced activity than three-argument verbs in the inferior frontal gyrus (IFG) of the left hemisphere. These results support (a) that syntactic processing of the verb's argument structure is related to verb itself and does not require a complete sentence and (b) that the IFG is crucially involved in the processing of this syntactic information.

The present fMRI study also confirmed our results of our previous MEG-study in that less complex, one-argument verb's elicited more pronounced activity than more complex, three-argument verbs. This contrasts the intuitive expectation, that the structurally more complex three argument verbs would lead to stronger brain responses compared to less complex one argument verbs. As an explanation, it might be assumed that the brain response may not reflect the structural complexity of the argument structure but rather, as to what extent the verb describes a complete event: Within the composition of words (including verbs) into a sentence, the "*completeness" *of the compositional representation of words may vary across the parsing process, being accompanied by a sequence or cascade of activation. Before the first word is presented, sentence processing (or verb retrieval) is 0% complete, and activation has not started. Upon word/verb presentation, the representation of a corresponding situation may be activation, and retrieval of the verb describing the situation is of highest impact. While the recognition of simple events is 50% complete (subject missing), the recognition of complex events may be less complete (33% for two place verbs, 25% for three place verbs; see [14]).

Stromswold *et al*., [11] found increased regional cerebral blood flow (rCBF) in Broca's area (particularly in the pars opercularis) when subjects judged the semantic plausibility of syntactically more relative to less complex sentences. In line with this finding, name-verb-pairs with one-argument verbs in the present study were more likely to reflect a complete event than three argument verbs. Posterior temporal activation has been demonstrated in verb generation tasks [9,15,16]. Similarly, the argument structure of isolated verbs was represented in the inferior temporal fusiform gyrus (BA 37) in the present study. This is in accordance to Bornkessel's view that this area (BAs 22/37/39) has an enhanced sensitivity for morphological information and the syntactic realization of the verb-based argument hierarchy.

Relative to the processing of isolated verbs, presentation of verbs in context of a proper name shifted the patter of activation to Brodman's Area 47. This is in line with higher activation in BA 47 for subject- than for object-initial sentences [4]. In the present study the name preceding the verb might have been interpreted as the subject of the beginning of a sentence followed by a grammatically correct inflected verb.

In their seminal studies, Petersen and colleagues [17,18] demonstrated the association between nouns and their related verbs: In a verb generation task, subjects articulated appropriate verbs to nouns that were presented either acoustically or visually. In a noun repetition task, subjects repeated acoustically presented nouns or articulated visually presented words. Relative to noun repetition, the verb generation task produced significantly more activation in a left inferior prefrontal region located at or near Brodmann area 47. This design can be compared to the present study, in which a proper name (a noun) was followed by a verb. One might speculate that the correspondence of the verb and the subject translates into the activity of the IFG. This again supports the significance of BA 47 for the coordination of words reflecting the strength of their (syntactic) connection.

Moreover, Kapur and colleagues [7] demonstrated that nouns were better recognized in a memory task when they were semantically processed, involving the activation of Brodmann Area 47 (left inferior prefrontal gyrus). Kapur et al. [19] conclude that "This finding suggests that the left inferior prefrontal cortex is the anatomical region involved in 'working with meaning', and that the activation does not reflect willed action, is not task-specific and is not attributable to the requirements of a spoken response."

The present study provides strong evidence that argument number dependent activation shifts from posterior, temporal areas to anterior, frontal areas when context is added. Rappaport and colleagues [20] assumed that a verb activates the semantic class as an intermediate representation. Similarly, Buckner et al. [21] suggested that anterior regions maintain or control access to higher-level semantic information. We argue that this information may resemble the dynamic representation of the sentence which integrates the subject into the argument structure of the verb leading to the representation of an event. Likewise, Wagner et al. [22] interpreted the left inferior prefrontal area as a semantic executive system that mediates on-line retrieval of long-term conceptual knowledge.

Syntactic [10,11,23-27] and semantic processing has consistently been found to activate left inferior frontal areas [7,28-35]. Specifically, a region in the anterior and ventral aspect of the inferior frontal gyrus (IFG, approximate BA47/10) has been identified as contributing to semantic processing, but also left middle temporal cortex [36,37] which was also active in the present study.

Thus, it is plausible that the IFG projects back to the temporal lobe to keep representations active. Such a structure would allow for lexical items to interact when coming in sequentially: The activation of typical fillers (subjects or obligatory objects) is supposed to facilitate on-line language processing. Reading is speeded up, when thematic roles were saturated (e.g. the subject was provided) during comprehension [38,39].

## Conclusion

In summary, visually presented verbs activate their argument structure in areas assigned to the biological lexicon. This information is used in the inferior frontal gyrus to integrate proceeding nouns into an event representation that is sequentially built up while new words come in. Thus, static parts of the representation stay active in the temporal lobe while dynamic parts may be processed in the IFG.

## Methods

### Subjects

20 healthy native German speaking subjects (mean age 27.1 ± 6.2 years, 11 females) were recruited for the study. All subjects were right-handed as assessed with the Edinburgh inventory [40]. Prior to the experiment, subjects were informed about measurement procedures and security issues and gave written informed consent. After the experiment, subjects received a bonus of 10 Euros. Ethical approval was granted by the ethics committee of the University of Konstanz.

### Experimental task and stimulus characteristics

A silent reading task was implemented during fMRI. Stimulation comprised four blocked experimental conditions [one-argument or three-argument verbs preceded by a name (N1; N3); one-argument or three argument verbs preceded by senseless letter strings that were matched to the names for length (V1; V3)] and a baseline condition (fixation cross).

Stimuli had been selected from a pilot study, in which 600 German verbs presented in third person, singular, present, active form with different argument-structures were pre-selected from the CELEX-database [41]. Verbs were selected to be as unambiguous as possible and having the lowest number of different argument structures [42,43]. Ten student volunteers were asked to generate a sentence to each verb. Non-obligatory adjuncts referring to time or space were not considered. A verb was included into the stimulus set, when more than 70% of the generated sentences included the argument structure of the central sense [44].

For each argument structure, 40 verbs were selected from the pool obtained in the pilot study [14]. Verbs were selected only, if they formed a meaningful beginning of a sentence with a name. One-argument verbs had a mean length of 7.6 letters (SD = 1.42), three-argument verbs a mean length of 7.6 letters (SD = 1.62). Both set were matched for frequency (mean: 2.2 per million words, [41]). Forty proper names were selected for the N-condition, while letter strings matched for length preceded the verbs in the V-conditions, in order to ensure physical comparability of the conditions.

Each condition was repeated 8 times and consisted of 5 consecutive trials. Each verb, name or letter string was shown only once to avoid repetition effects. The sequence of blocks was fixed, while presentation of stimuli within blocks was random. Two different sequences of blocks were designed and counterbalanced across subjects. Stimuli were presented by a visor (VisuaStim, Resonance Technology, Inc.) in the middle of the screen. Subjects were instructed to silently read the stimuli.

### Scanning parameters

A 1.5 Tesla Philips Intera MR-System equipped with Power Gradients Scanning was used. For functional scanning, a T2*-weighted Fast-Field Echo, Echo-Planer-Imaging (FFE-EPI) sequence utilizing a parallel scanning technique (SENSE; [45]) was used. Images were acquired in transversal orientation parallel to the AC-PC line. Each dynamic volume consisted of 36 slices measured in interleaved acquisition order with a thickness of 4.5 mm each. In plane resolution was 2.9 × 2.9 × 3.5 mm with a squared Field-of-View at a size of 230 mm (acquisition matrix 80 × 80 voxels). A whole-head scan was assessed every 3 sec (TR), the overall sequence comprising 200 continuously acquired volumes. Eight dummy scans serving for T1-equilibration at the beginning of the experimental session were discarded from data analysis.

***Functional MRI post-processing ***was accomplished with the Statistical Parametric Mapping (SPM2, Wellcome Department of Cognitive Neurology, London, UK). Pre-processing included correction for slice-time differences and spatial alignment to the first volume of the image series to adjust for head movements during the course of the experiment. Subsequently, functional volumes were normalized to MNI standard stereotactic space and smoothed with a Gaussian Kernel of 8 × 8 × 9 mm full-width-at-half-maximum (FWHM).

Pre-processed data were submitted to statistical analysis implementing the General Linear Model (GLM). The corresponding design matrix comprised the 5 covariates-of-interest representing the experimental conditions' onsets, the duration of the different presentation epochs, and no-interest-covariates (modelled response functions' time and dispersion derivatives and six movement parameters obtained during realignment). Before estimating the modelled regressors, a high pass filter with a cut-off period of 128 sec was applied to the data. Following estimation of the overall model, planned contrasts-of-interest were calculated for each subject, including the experimental conditions (V1/V3/N1/N3) and the fixation baseline. Moreover, specific contrasts comprised the conditions: V1 > V3 and N1 > V3 and the inverse contrasts, in order to elucidate differential activity associated with the processing of the different argument structures and the effect of filling the argument structure. Further contrasts included comparisons between isolated verbs and such that were preceded by a proper name (N1 > V1 and N3 > V3), verbs with and without context (N1/3 > V1/3).

For the group analysis a random effect model included these contrasts of all subjects. Maximally activated voxels within significant clusters are reported (cluster threshold p < .05 family wise error-corrected, FWE, cluster extent k>20; voxel threshold p < .001 uncorrected). Significant voxels within clusters were anatomically localized with the Talairach Demon software [46]. For graphical display activated areas were projected onto a template of a standard MNI brain.

## Authors' contributions

RA and BR proposed the general research question. RA, MM, TF, and JO designed the experiment. MM and TF accomplished data collection, analyzed the data and contributed the methods and results sections. RA drafted the initial version of manuscript, all authors contributed to the discussion of the results. All authors read and approved the final manuscript.

## Supplementary Material

Additional file 1 **Basic contrasts (V1/V3/N1/N3 > Fixation). **The data provided describes details of the activity patterns for the following contrasts: V1 > Fixation, V3 > Fixation, N1 > Fixation, N3 > Fixation Click here for file
