# Supplementary material for: The representation of the verb's argument structure as disclosed by fMRI
Source: BMC Neurosci. 2009 Jan 15;10:3. doi: 10.1186/1471-2202-10-3 (PMC2632636; doi:10.1186/1471-2202-10-3)
Supplement: Additional file 1 — Basic contrasts (V1/V3/N1/N3 > Fixation). The data provided describes details of the activity patterns for the following contrasts: V1 > Fixation, V3 > Fixation, N1 > Fixation, N3 > Fixation [file 1471-2202-10-3-S1.doc]

**Additional file 1**

|  | **Isolated one-argument verbs (V1)** | | | | | | |  | **Isolated three-argument verbs (V3)** | | | | | | |
| --- | --- | --- | --- | --- | --- | --- | --- | --- | --- | --- | --- | --- | --- | --- | --- |
| **hemi** | **anatomic**  **structure** | **BA** | **k** | **Z** | **x** | **y** | **z** | **hemi** | **anatomic**  **structure** | **BA** | **k** | **Z** | **x** | **y** | **z** |
| L/R  R | Middle/inferior occipital  Fusiform gyrus | 18  19 | 6143 | 6.1  5.6 | -42  40 | -76  -73 | -8  -15 | R | Middle/inferior occipital  Fusiform gyrus | 18  19 | 2232 | 5.5  5.1 | 40  40 | -86  -71 | -1  -15 |
| L | Precentral gyrus  Middle frontal | 4  6/9 | 289 | 4.3  4.2 | -48  -42 | -8  9 | 43  33 | L | Inferior occipital | 18/17 | 2513 | 5.4 | -36 | -86 | -6 |
| L | Superior parietal * | 7 | 44 | 4.2 | -28 | -56 | 47 | L | Inferior frontal * | 47 | 45 | 4.0 | -48 | 27 | -5 |
| L | Middle temporal | 21/22 | 137 | 4.1 | -65 | -29 | -2 | L | Middle temporal * | 21 | 45 | 3.7 | -61 | -31 | -3 |
| L | Superior temporal * | 13 | 38 | 3.8 | -50 | -44 | 21 |  |  |  |  |  |  |  |  |
|  |  |  |  |  |  |  |  |  |  |  |  |  |  |  |  |
|  | **One-argument verbs in context (N1)** | | | | | | |  | **Three-argument verbs in context (N3)** | | | | | | |
| **hemi** | **anatomic**  **structure** | **BA** | **k** | **Z** | **x** | **y** | **z** | **hemi** | **anatomic**  **structure** | **BA** | **k** | **Z** | **x** | **y** | **z** |
| R/L  L | Middle occipital  Cuneus | 18  17 | 4932 | 5.7  5.6 | 28  -16 | -93  -95 | 5  -2 | R | Inferior/middle occipital | 18/19 | 2227 | 4.9 | 40  36 | -86  -87 | -1  10 |
| L | Inferior frontal | 47 | 259 | 4.5 | -51 | 21 | -4 | L | Inferior occipital  Fusiform gyrus | 18/19  18 | 2420 | 4.8  4.8 | -38  -42 | -88  -72 | -6  -10 |
| L | Middle temporal | 21/20 | 401 | 4.0 | -57 | -24 | -7 | L | Inferior temporal  Middle temporal | 20  21 | 324 | 4.3  4.1 | -55  -51 | -9  -29 | -18  -2 |
| L | Middle frontal * | 9 | 41 | 3.9 | -53 | 17 | 32 | L | Medial/inferior frontal * | 11/47 | 42 | 3.9 | -4 | 53 | -18 |
|  |  |  |  |  |  |  |  | L | Parahippocampal gyrus * | 35/28 | 66 | 3.8 | -16 | -28 | -9 |

L=left, R=Right; hemi=hemisphere; BA=Brodman Area; k=cluster extent; x/y/z=Talairach-coordinates

Z-values for maximally activated voxels within significant clusters

* only significant at an uncorrected cluster level of p< .05
